# Combatting AMR: photoactivatable ruthenium(ii)-isoniazid complex exhibits rapid selective antimycobacterial activity†
†Electronic supplementary information (ESI) available: Experimental details include materials, instrumentation, synthesis and characterization, photoactivity against bacteria, transient electronic absorption spectroscopy and computational analysis. Table S1–S5 show the data of X-ray structures and DFT analysis, Fig. S1–S12 give X-ray crystal structures and dark stability of the compound, the extinction coefficients for *cis*-[Ru(bpy)_2_(INH)_2_]^2+^, HR-MS peaks for the photoproduct, the stability of the photoproduct, ^1^H NMR spectrum and HPLC analysis upon photoirradiation, photoactivity against bacteria and the pictures of DFT analysis. CCDC 1474453. For ESI and crystallographic data in CIF or other electronic format see DOI: 10.1039/c6sc03028a



**DOI:** 10.1039/c6sc03028a

**Published:** 2016-08-30

**Authors:** Nichola A. Smith, Pingyu Zhang, Simon E. Greenough, Michael D. Horbury, Guy J. Clarkson, Daniel McFeely, Abraha Habtemariam, Luca Salassa, Vasilios G. Stavros, Christopher G. Dowson, Peter J. Sadler

**Affiliations:** a Department of Chemistry , University of Warwick , Gibbet Hill Road , Coventry CV4 7AL , UK . Email: P.J.Sadler@warwick.ac.uk; b School of Life Sciences , University of Warwick , Gibbet Hill Road , Coventry CV4 7AL , UK . Email: C.G.Dowson@warwick.ac.uk; c CIC biomaGUNE , Paseo de Miramón 182 , Donostia-San Sebastián , 20009 , Spain; d Kimika Fakultatea , Euskal Herriko Unibertsitatea and Donostia International Physics Center (DIPC) , P.K. 1072 , Donostia-San Sebastián , 20080 , Spain; e Ikerbasque , Basque Foundation for Science , Bilbao , 48011 , Spain

## Abstract

The novel photoactive ruthenium(ii) complex *cis*-[Ru(bpy)_2_(INH)_2_][PF_6_]_2_ (**1**·2PF_6_, INH = isoniazid) was designed to incorporate the anti-tuberculosis drug, isoniazid, that could be released from the Ru(ii) cage by photoactivation with visible light. In aqueous solution, **1** rapidly released two equivalents of isoniazid and formed the photoproduct *cis*-[Ru(bpy)_2_(H_2_O)_2_]^2+^ upon irradiation with 465 nm blue light. We screened for activity against bacteria containing the three major classes of cell envelope: Gram-positive *Bacillus subtilis*, Gram-negative *Escherichia coli*, and *Mycobacterium smegmatis in vitro* using blue and multi-colored LED multi-well arrays. Complex **1** is inactive in the dark, but when photoactivated is 5.5× more potent towards *M. smegmatis* compared to the clinical drug isoniazid alone. Complementary pump-probe spectroscopy measurements along with density functional theory calculations reveal that the mono-aqua product is formed in <500 ps, likely facilitated by a ^3^MC state. Importantly, complex **1** is highly selective in killing mycobacteria *versus* normal human cells, towards which it is relatively non-toxic. This work suggests that photoactivatable prodrugs such as **1** are potentially powerful new agents in combatting the global problem of antibiotic resistance.

## Abstract 2

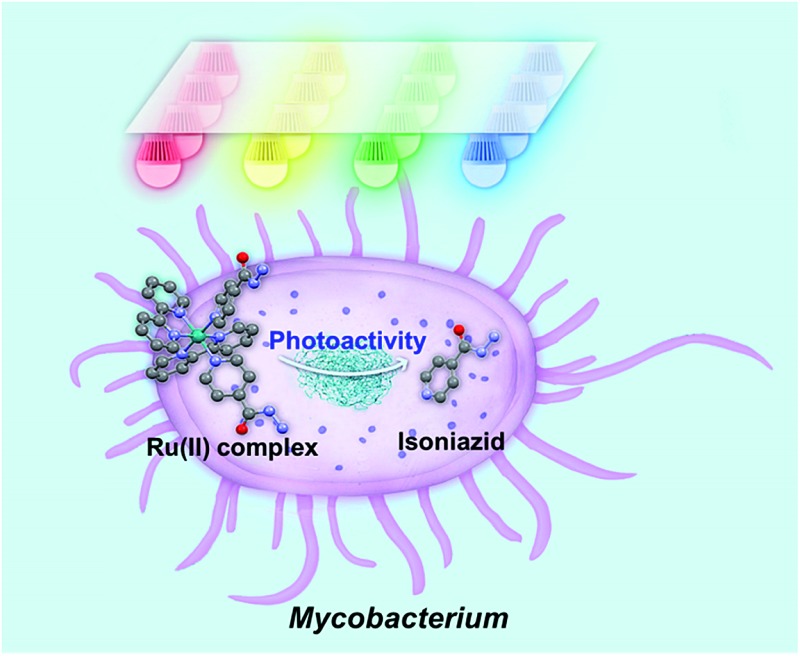
The Ru(ii) complex, *cis*-[Ru(bpy)_2_(INH)_2_]^2+^ is inactive in the dark but highly active towards mycobacteria on photoactivation with visible light when it releases the antituberculosis drug isoniazid (INH).

## Introduction

Antibacterial resistance (AMR) is a complex problem that contributes to health and economic losses worldwide. Resistance to antimicrobial therapies reduces the effectiveness of current drugs, leading to increased morbidity, mortality, and health care expenditure. Because globalization increases the vulnerability of any country to diseases occurring in other countries, resistance presents a major threat to global public health.^1^ Mycobacteria, such as *Mycobacterium tuberculosis* and *Mycobacterium ulcerans*, are resistant to many antibiotics and their cell-wall structure is believed to be largely responsible for the wide range of resistance phenotypes.^2^ *M. tuberculosis* is a major cause of death worldwide, and a formidable enemy infecting one third of the world. In 2014, there were an estimated 9.6 million new TB infections worldwide.^1*a*^ By comparison, *M. ulcerans* causes Buruli ulcer (BU) and is the third most important mycobacterial disease after tuberculosis and leprosy worldwide. Furthermore, WHO reports that it is endemic in West Africa as well as China and Australia, and its geographic range is increasing.^3*a*^

Frequent and inappropriate use of antibiotics to treat TB has resulted in the emergence of multidrug-resistant *M. tuberculosis* (MDR TB) and extensive drug-resistant *M. tuberculosis* (XDR TB) to front-line (isoniazid and rifampin) and second-line drugs (amikacin, kanamycin and capreomycin).^1*a*^ WHO recommendations for BU treatment, as with TB treatment, involve combination therapy (rifampicin, streptomycin or rifampicin, clarithromycin). However, as therapy is rolled out, the emergence of resistance is an inevitable outcome. Ineffective treatment often requires surgery, limb amputation, and sometimes results in death. Therefore, there is an urgent need to develop new drugs and innovative strategies to tackle mycobacterial infections.

Considering the time-consuming discovery process and the high mutation rate of *Mycobacterium* spp. to current antibiotics, novel mechanisms for attack on bacteria are essential. In recent years, photoactive therapy to combat cancer or bacterial infection has attracted increasing interest.^4^ Photoactive antimicrobial therapy includes two kinds of therapy, photodynamic antimicrobial therapy (PDAMT)^5^ and photorelease antimicrobial therapy (PRAMT).^6^ PDAMT and PRAMT are promising strategies for treating surface bacterial infections, especially patients with skin infection, scar tissues, burn infections, leg ulcers in diabetes patients, acne infection, and for sterilization of some surfaces.^7^ PDAMT utilizes light and oxygen in combination with a photosensitizer (PS).^8^ The ground state (GS) of the PS absorbs visible light to reach a triplet excited state (ES) *via* intersystem crossing and then generates the reactive oxygen species (ROS) ^1^O_2_, which is highly toxic and can cause non-specific damage to bacterial cell components. PRAMT can control the time and place of release of a therapeutic agent to achieve targeted therapy and reduce systemic toxicity to host tissues.

**Scheme 1:**
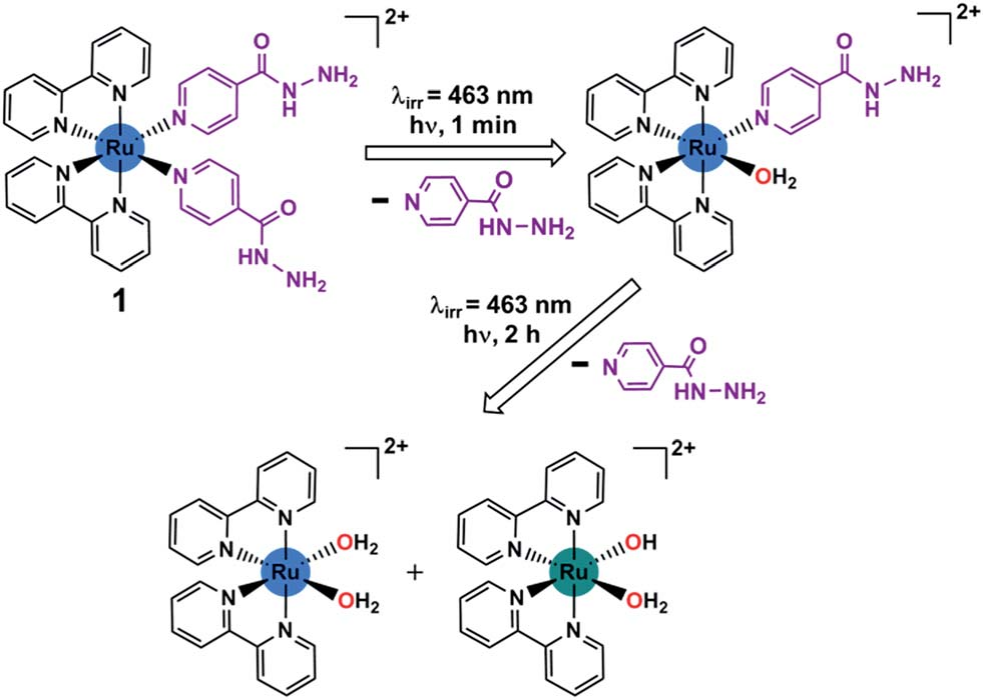
Stepwise photoactivation of the antibacterial prodrug *cis*-[Ru(bpy)_2_(INH)_2_]^2+^ (**1**). Ru(ii) is blue, and Ru(iii) is green.

Ruthenium(ii) bipyridyl complexes can behave as efficient photoactivatable prodrug delivery systems, as shown by *e.g.* Etchenique,^9^ Turro,^10^ Glazer^11^ and Gasser.^12a,20^ Recently, some Ru(ii) complexes have been studied for antibacterial activity.^12^ Ru(ii) polypyridyl complexes containing extended aromatic ligands are active against Gram-positive *B. subtilis* and *S. aureus*, but inactive against Gram-negative *E. coli*.^13^ [Ru(bpy)_2_(NPDA)]^2+^ (NPDA = *N*-phenyl-substituted diazofluorene) is active against methicillin-resistant *S. aureus* (MRSA) and generates ROS to kill MRSA.^14^ Some dinuclear Ru(ii) complexes have been studied, where rigid linkers between metal centers show lower activity against *S. aureus* and *E. coli* compared to flexible linkers.^15^ The activity appeared to be linked to lipophilicity, as increased lipophilicity results in increased penetration through cell membranes and therefore increased uptake.^16^ Another strategy used in the design of antibacterial agents is to attach an existing organic antibacterial agent to a Ru(ii) center, for example, attachment of a β-lactam to a cyclopentadienyl ligand or using ofloxacin as a chelating ligand.^17^ This strategy can be used either in an attempt to overcome resistance, or to achieve a potential synergy between the metal and the antibacterial agent. Currently there are few examples of Ru(ii) complexes that are being developed as anti-mycobacterial agents. Complexes containing phosphine, diimine and picolinate groups have shown promising activity against *M. tuberculosis*.^18^ They have shown better activity than isoniazid against *M. tuberculosis* and retain activity against an isoniazid-resistant strain.^18b,c^ Here we investigate the feasibility of using a potentially transformative strategy to develop photoactive anti-mycobacterial drugs. We have designed a photoactivatable Ru(ii) complex that can release an antimicrobial agent upon light irradiation. The photoactivatable complex is based on *cis*-[Ru(bpy)_2_(INH)_2_]^2+^, where bpy is bipyridine and INH is isoniazid (anti-tuberculosis compound). The aim of this study is to release the biologically active isoniazid ligand by photoactivation and form a ruthenium(ii) aqua species (Scheme 1). Encouragingly we found that complex **1** is selectively and potently active upon irradiation towards the mycobacterium *M. smegmatis*.

## Results and discussion

### Chemical synthesis and structure

To synthesize **1**·2PF_6_, *cis*-[Ru(bpy)_2_Cl_2_] and isoniazid (INH) were heated in degassed water under nitrogen at 353 K for 6 h. The solution was allowed to cool to ambient temperature and NH_4_PF_6_ was added. The precipitate formed was collected by filtration and washed sequentially with cold water and diethyl ether. Crystals suitable for X-ray crystallography (Fig. S1a†) were obtained by slow diffusion of 1,4-dioxane into a saturated acetonitrile solution of **1**·2PF_6_ at room temperature. The crystal structure showed the presence of both enantiomers (Δ and Λ) in the unit cell (Fig. S1†). The crystallographic data and selected bond lengths and angles are given in Table S1 and S2.† The unit cell contains pairs of enantiomers connected *via* a CH–π interaction between CH of bpy from the Δ-complex and the π system of INH from the Λ-complex (Fig. S1b†). The CH(bpy)-centroid(INH) distance is 3.176 Å and the angle between the ring planes is 81.76°. There is a small twist angle of 3.29° between the pyridine rings of the bpy ligand. Each hydrazide group on the INH ligand forms a hydrogen-bonded dimer that links symmetry-related enantiomers forming an infinite zigzag chain. This is composed of a reciprocal interaction between the hydrazide amide NH and the amine NH2 of the hydrazide of a symmetry-related INH ligand related by an inversion centre with an H-bond distance of N–H···N 2.199 Å (Fig. S1c†).

### Photorelease analysis

**Fig. 1:**
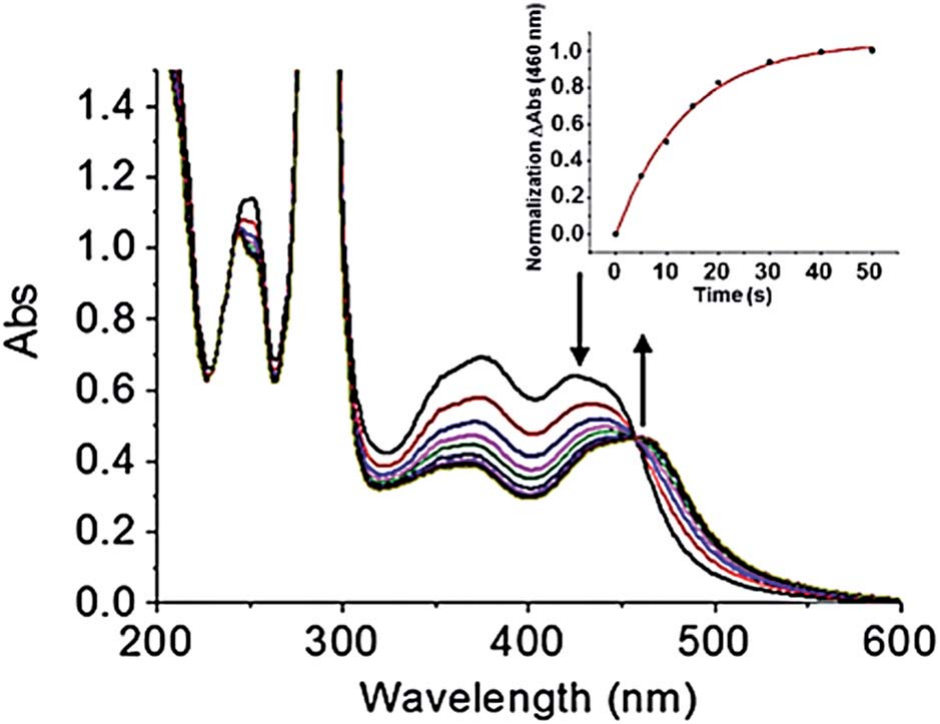
UV-visible spectra of 40 μM *cis*-[Ru(bpy)_2_(INH)_2_]^2+^ on photoirradiation with blue light (*λ*_irr_ = 465 nm, 20 mW cm^–2^), with kinetic trace showing formation of the photoproduct (inset graph).

Prior to investigating the photoactivity of the complex, it is imperative to understand how the complex behaves in the dark. The UV-visible spectra showed no appreciable change when stored in aqueous solution over a 6 h period in the dark (Fig. S2†). It was stable in the dark for the duration of the experiment. When an aqueous solution (40 μM) of *cis*-[Ru(bpy)_2_(INH)_2_]^2+^ was exposed to blue light (*λ*_irr_ = 465 nm, 20 mW cm^–2^) at 298 K for <1 min, there were changes in the UV-visible spectra (Fig. 1a). There was a red shift of the major absorption band whereby the peak at ∼420 nm decreased and the peak at ∼460 nm increased in intensity, producing an isosbestic point at ∼450 nm. The presence of an isosbestic point suggested that a stoichiometric reaction was occurring. In <1 min the production of the photoproduct reached a plateau. The increase of the peak at ∼460 nm with time followed a single exponential relationship, as illustrated in Fig. 1, inset, which plots the change in photoproduct absorbance (ΔAbs), at 460 nm, *versus* time. Unsurprisingly, the time taken for the photoproduct to plateau in ΔAbs was dependent on the wavelength of activation, given the change in extinction coefficient (Fig. S3†). Photoactivity was observed upon exposure to radiation between 440 and 560 nm, with the photoproduct ΔAbs taking *ca.* 40 s to plateau upon 465 nm light irradiation (Fig. 1, inset graph). There was no measurable photoactivity upon irradiation with red light (*λ*_irr_ = 610 nm).

### Photoproduct analysis

In order to characterise the photoirradiation products fully, liquid chromatography coupled to high resolution mass spectrometry (LC-HRMS) was employed. An aqueous solution (40 μM) of *cis*-[Ru(bpy)_2_(INH)_2_]^2+^ was photoirradiated with blue light (*λ*_irr_ = 465 nm, 20 mW cm^–2^) for 1 min, 30 min, 1 h and 2 h. The chromatogram at each timepoint of photoirradiation and corresponding peaks in the high resolution mass spectrum of each detected product peak are shown in Fig. S4† and Table 1, respectively. With no photoirradiation, there was only one peak in the chromatogram (peak A) with a retention time of 7.4 min, corresponding to the starting material *cis*-[Ru(bpy)_2_(INH)_2_]^2+^. After 1 min of photoirradiation, peak A was no longer present and there were two new peaks at 8.1 min (peak B) and 3.2 min (peak C), which correspond to *cis*-[Ru(bpy)_2_(INH)(H_2_O)]^2+^ and free INH, respectively. With increase in time of photoirradiation, peak B decreased in intensity and peak C increased in intensity. After 30 min of photoirradiation, there were two new peaks at 8.9 min (peak D) and 6.7 min (peak F), which correspond to the di-aqua photoproducts *cis*-[Ru(bpy)_2_(H_2_O)_2_]^2+^ and *trans*-[Ru(bpy)_2_(H_2_O)_2_]^2+^, respectively. After 1–2 h photoirradiation, the aqua/hydroxido Ru^III^ complex *trans*-[Ru(bpy)_2_(H_2_O) (OH)]^2+^ (peak E at 4.3 min) was detected; the mass-to-charge ratio and isotopic models for peak E are consistent with the formation of a Ru(iii) species (Fig. S5†).

**Table 1:** Photoirradiation products detected and characterized by LC-HRMS (according to Fig. S4)

Peak	RT (min)	MS (*m*/*z*)	Formula calculated *m*/*z*	Error (ppm)
A	7.4	344.0794	*Cis*-[Ru^II^(bpy)_2_(INH)_2_]^2+^	0.87
			C_32_H_30_N_10_O_2_Ru: 344.0797	
B	8.1	284.5553	*Cis*-[Ru^II^(bpy)_2_(INH)(H_2_O)]^2+^	0.70
			C_26_H_25_N_7_O_2_Ru: 284.5555	
C	3.2	128.0663	[INH + H]^+^	0.72
			C_6_H_7_N_3_O: 138.0662	
D	8.9	225.0306	*Cis*-[Ru^II^(bpy)_2_(H_2_O)_2_]^2+^	2.67
			C_20_H_20_N_4_O_2_Ru: 225.0312	
E	4.3	224.5273	*Trans*-[Ru^III^(bpy)_2_(H_2_O) (OH)]^2+^	0.00
			C_20_H_19_N_4_O_2_Ru: 224.5273	
F	6.7	225.0304	*Trans*-[Ru^II^(bpy)_2_(H_2_O)_2_]^2+^	1.03
			C_20_H_20_N_4_O_2_Ru: 225.0312	

The photoproducts at the different photoirradiated times all appeared to be stable in solution, as there were no changes in the UV-visible spectrum after 1 h of incubation in the dark at 298 K (Fig. S6†). In addition, a solution of complex **1** in D_2_O and photoirradiated with a blue LED for 1 min was monitored by ^1^H-NMR. After 1 min photoirradiation, the peaks assignable to the starting material *cis*-[Ru(bpy)_2_(INH)_2_]^2+^ decreased in intensity and two new sets of peaks appeared. The first set of 2 peaks (8.68 ppm and 7.69 ppm) are due to the released free ligand (INH), while the second set of peaks is assigned to the photoproduct *cis*-[Ru(bpy)_2_(INH)(D_2_O)]^2+^ (Fig. S7†).

### Photoactive studies on bacteria and mammalian cell

**Fig. 2:**
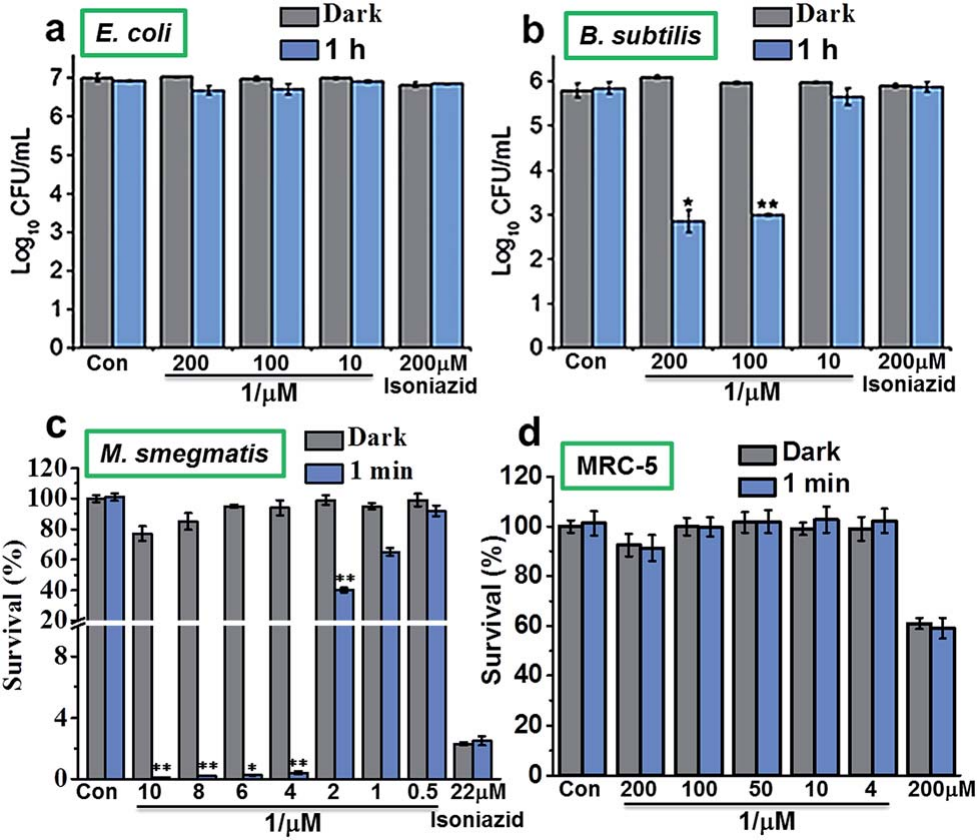
Dose-response for INH alone or *cis*-[Ru(bpy)_2_(INH)_2_]^2+^ (**1**) when incubated with the 3 classes of bacteria (a) Gram-negative *E. coli*, (b) Gram-positive *B. subtilis*, (c) *M. smegmatis* and (d) normal human lung fibroblast cell line MRC-5 in the dark (gray bars) or photoirradiated (blue bars) using the 96-array blue LED (*λ*_irr_ = 465 nm, 20 mW cm^–2^) for various times at 298 K. The survival at each photoirradiation timepoint was compared to the light control in the absence of the complex, while the survival for the complex in the dark was compared to the dark control in the absence of complex. *p* values were calculated by comparing the light samples to the dark samples and are labelled as follows, *p* ≤ 0.05 = *, *p* ≤ 0.01 = **.

The photoactivity of complex **1** against Gram-positive, Gram-negative and mycobacteria (*B. subtilis*, *E. coli* and *M. smegmatis*, respectively) *in vitro* was compared using 96-array blue LED (*λ*_irr_ = 465 nm, 20 mW cm^–2^) and 32-array multi-coloured LED (*λ*_irr_ = 465 nm (blue), 520 nm (green), 589 nm (yellow) and 625 nm (red), 5 mW cm^–2^) irradiation. The designs for the LED arrays are shown in Fig. S8†. Firstly, *cis*-[Ru(bpy)_2_(INH)_2_]^2+^ was tested against Gram-positive *B. subtilis* and Gram-negative *E. coli* models in the dark or photoirradiated for 2 h using the 96-array blue LED. The number of colony-forming units (CFU mL^–1^) was measured. For *E. coli*, the log_10_(CFU mL^–1^) was found to be ∼7 for all concentrations (200 μM, 100 μM and 10 μM) both in the dark and upon photoirradiation. Additionally, log_10_(CFU mL^–1^) for the control (cells not exposed to **1**) and for isoniazid alone (200 μM) was also ∼7 both in the dark and after photoirradiation (Fig. 2a). For *B. subtilis* and concentrations of 200 μM and 100 μM **1**, log_10_(CFU mL^–1^) was ∼6 in the dark, and decreased to ∼3 when photoirradiated (Fig. 2b). Log_10_(CFU mL^–1^) for the control and isoniazid alone (200 μM) was ∼6 for both dark incubation and after photoirradiation.

The photoactivity of **1** towards *Mycobacterium smegmatis* was then investigated. When *M. smegmatis* was incubated with *cis*-[Ru(bpy)_2_(INH)_2_]^2+^ in the dark, the survival was 80%, 71% and 65% for 10 μM, 30 μM and 50 μM complex **1**, respectively. However, after 1 min, 30 min, 1 h and 2 h photoirradiation, the survival was <1% at all these concentrations (Fig. S9†). The MIC of **1** after photoirradiation for 1 min (Fig. 2c) was found to be 4 μM. The minimum inhibitory concentration (MIC) for isoniazid alone is comparable to literature values (*c.f.* 29 μM).^19^ However, since the survival was >90% at a concentration of 4 μM in the dark, the results suggest that the complex has a low toxicity in the dark and high photocytotoxicity.

**Fig. 3:**
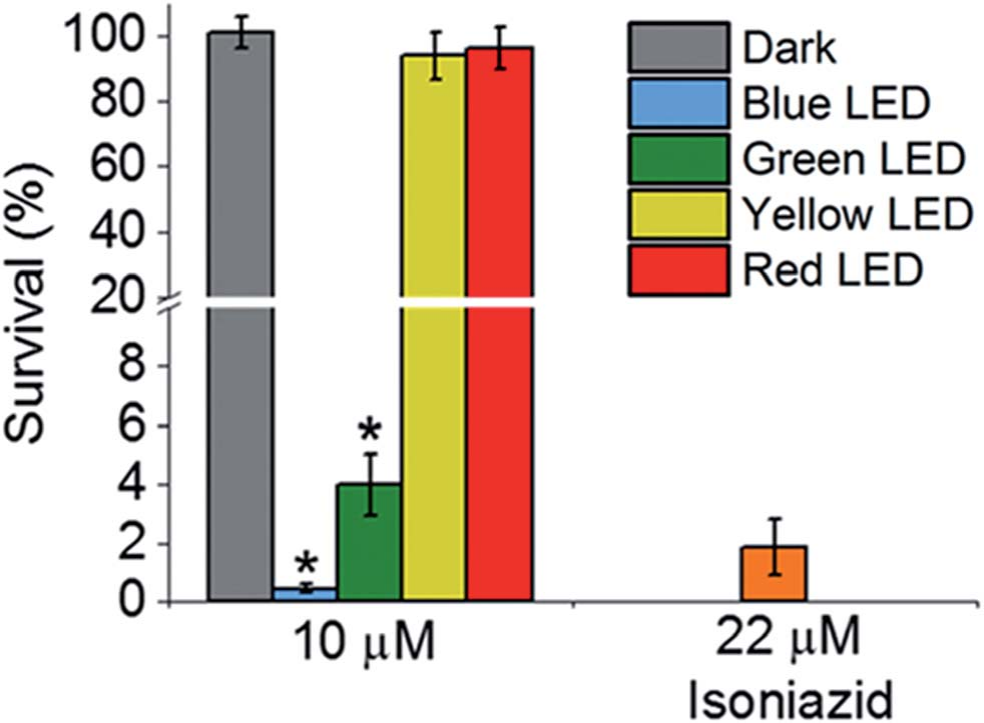
Activity of *cis*-[Ru(bpy)_2_(INH)_2_]^2+^ against *M. smegmatis* when incubated in the dark using the 32-array of multi-colored LEDs (*λ*_irr_ = 465 nm, 520 nm, 589 nm and 625 nm, 5 mW cm^–2^) for 30 min. *p* values were calculated by comparing the light and dark samples and are labelled as follows, *p* ≤ 0.05 = *.

Then we determined the toxicity of **1** towards the (normal) human lung cell line MRC-5. The complex was non-toxic, with survival levels of >90% at all concentrations tested, both in the dark and upon irradiation with blue light (Fig. 2d). However, when MRC-5 lung cells were incubated with 200 μM isoniazid itself, the cell viability was only ∼60%. This suggests that complex **1** has a high selectivity for *M. smegmatis* bacteria *versus* normal human cells, a highly desirable property for a new antibiotic. When *M. smegmatis* was photoirradiated using the 32-array of multi-colored LEDs for 30 min in the absence of the complex, the survival was >90% for all wavelengths (blue, green, yellow, and red; Fig. S10†). The dark survival of *M. smegmatis* incubated with the complex was >90%. After *M. smegmatis* was incubated with *cis*-[Ru(bpy)_2_(INH)_2_]^2+^ in the dark for 1 h and the samples were then photoirradiated for 30 min, survival increased as the wavelength of photoirradiation increased: blue 0.3%, green 4%, yellow 94%, and red 96% (Fig. 3).

### TEAS, DFT and TD-DFT analysis

To further understand the photoactivity of **1**, and in particular the initial stage of photoactivation, we performed transient electronic (UV-vis) absorption spectroscopy (TEAS). This technique has previously provided a detailed understanding of the pathways involved in photoactivation of this type of Ru^II^ complex.^21,22^ Efficient population of a dissociative triplet metal-centered (^3^MC) state is key to generating high quantum yields of a penta-coordinate intermediate (PCI) species, which in turn may form the target species: a mono-aqua photoproduct. We showed that photoactivation of *cis*-[Ru(bpy)_2_(NA)_2_]^2+^ to *cis*-[Ru(bpy)_2_(NA) (H_2_O)]^2+^ (NA = nicotinamide) occurs with a quantum yield ≥0.36, all within a timeframe of ∼400 ps.^22^

**Fig. 4:**
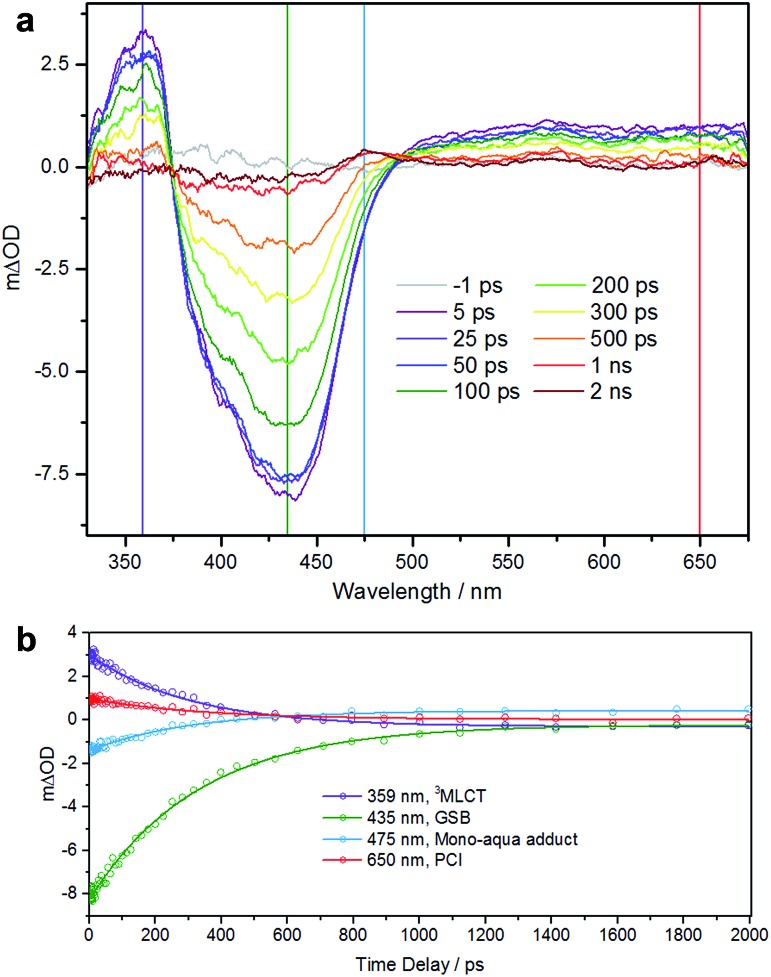
(a) TAS of an 890 μM aqueous solution of **1** over pump-probe time delays of –1 ps to 2 ns following excitation at 340 nm. Vertical lines correspond to positions of kinetic traces. (b) Kinetic traces and corresponding fits for the four features as described in the main text.

We performed TEAS to monitor the production of the mono-aqua photoproduct and extract the timescales involved. Details of the TEAS setup were as reported^22^ and as described in the ESI.† An aqueous solution of **1** flowing through a 950 μm path length flow cell was photoexcited using a 340 nm (650 mW, 2 mJ cm^–2^) pump pulse and probed using a white light continuum at pump-probe time delays ranging from –1 ps to 2 ns. The resulting transient absorption spectra (TAS) are shown in Fig. 4a for selected time delays. The TAS at time delays <500 ps comprise three distinct regions. From previous studies^23^ and related literature,^24^ it is known that the feature at ∼360 nm is the absorption of the ^3^MLCT state which is produced very shortly after excitation (<100 fs) following intersystem crossing from the initially populated ^1^MLCT state. Comparison with the steady-state UV-visible absorption spectra (see Fig. 1a) reveals the feature at ∼435 nm to be a ground state bleach. The third feature, a broad absorption centred around 650 nm contains some contribution from the ^3^MLCT state, but is mostly attributable to the PCI.^22^ For TAS at time delays ≥500 ps a fourth feature is clearly visible ∼475 nm. Comparison, once again with the steady-state UV-visible absorption spectra indicates that this is absorption by the mono-aqua (*cis*-[Ru(bpy)_2_(INH)(H_2_O)]^2+^) photoproduct.

To obtain lifetimes of these features, we gathered kinetic traces (5 nm-wide integrations around the indicated wavelength) and fitted them with mono exponential functions. These fits are shown in Fig. 4b. The summary of obtained lifetimes is as follows: *τ*_3MLCT_ = 207 ± 10 ps, *τ*_GSB_ = 231 ± 7 ps, *τ*_mono-aqua_ = 211 ± 13 ps and *τ*_PCI_ = 236 ± 30 ps, the subscript pertaining to the associated species. It should be noted that due to the severity of the spectral overlap of the TAS features, there is contamination of signal in each of the kinetic traces (from neighbouring features) and the extracted lifetimes will have additional error that has not been accounted for. A more rigorous treatment of these data necessitates a target or global analysis to deconvolute the TAS.^22^

However the main objective of the present work was to determine a timescale for the appearance of the mono-aqua photoproduct, which we propose corresponds to <500 ps. We also note that there is no evidence for the di-substituted photoproduct, which is unsurprising, given that this is a sequential process requiring the mono-aqua photoproduct to absorb an additional photon.

Computational chemistry studies confirmed that *cis*-[Ru(bpy)_2_(INH)_2_]^2+^ displays a photochemical behaviour typical of [Ru(bpy)_2_(L)_2_]^2+^ scaffolds (where L = monodentate N-ligand, Fig. S11†), in agreement with our TEAS characterization and previous experimental and theoretical work performed on analogous systems.^21,25^

**Fig. 5:**
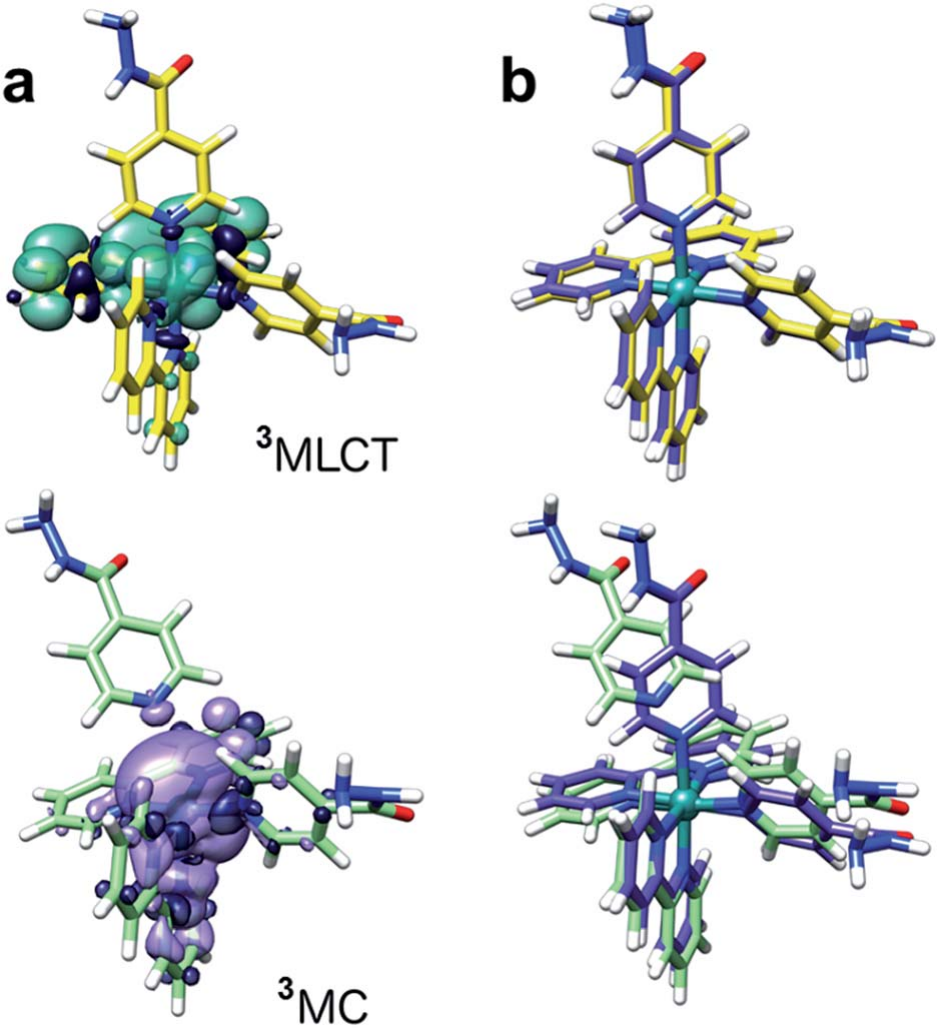
DFT-optimized structures of the ^3^MLCT (yellow) and ^3^MC (light green) states of **1** with (a) spin density surfaces (isovalue 0.0004) and (b) their superimposition with the ground-state structure (violet) of the complex.

Singlet–singlet transitions calculated by time-dependent density functional theory (TD-DFT) on the ground-state geometry of **1** show the lowest energy bands (300–500 nm) in the UV-visible spectrum and are predominantly of MLCT character (Ru-bpy), whereas absorption peaks above 300 nm originate from transitions with a significant ligand (bpy) component (Table S4 and Fig. S12†). Dissociative metal-centred (MC) singlet transitions are accessible at *ca.* 350 nm and involve Ru–N(INH) antibonding orbitals with strong Ru(d) character (Fig. S13†). Analysis of triplet geometries further confirms the prototypical behaviour of **1**. Two low-lying triplets were optimized, corresponding to the ^3^MLCT and ^3^MC states (spin density surfaces, Fig. 5 and Table S5†). The latter is 0.57 eV more stable than the ^3^MLCT state, hence it can be effectively populated upon light excitation. Furthermore, one of the Ru–N(INH) bonds is broken (3.79 Å) in the ^3^MC state which may correspond to the PCI detected by TEAS.

## Conclusion

In conclusion, the antimicrobial activity of the photoactive complex *cis*-[Ru(bpy)_2_(INH)_2_]^2+^ was explored. Firstly, UV-visible spectroscopy and HR-LCMS studies showed that irradiation with blue or green light rapidly leads to the release of the antibacterial ligand INH in aqueous media together with formation of mono- and di-aqua adducts. Secondly, *cis*-[Ru(bpy)_2_(INH)_2_]^2+^ was inactive against the Gram-negative bacterium *E. coli* and showed only slight activity once photoirradiated with blue light for 2 h against the Gram-positive bacterium *B. subtilis* at concentrations of 100 μM and 200 μM. The high photoactivity of **1** was evident from its high potency towards the mycobacterium *M. smegmatis* with an MIC value of 4 μM after only 1 min of photoirradiation with blue light. This was a 5.5× increase in potency compared to isoniazid alone. The survival of the bacteria incubated with the complex in the dark is >80% at the MIC value, showing that the active ligand (isoniazid) was successfully released only upon photoirradiation. Activation of the complex in cells was optimum for blue (465 nm) and green (520 nm) light irradiation, but no activation was observed with yellow or red light. Complementary pump-probe spectroscopy measurements have shown that the mono-aqua photoproduct is formed in <500 ps, potentially mediated by a ^3^MC state, which, as DFT calculations show, has antibonding character along the Ru–INH bond. If longer wavelengths are required for deeper tissue penetration, then activation may be achievable with two-photon methods, which are currently being explored in our laboratory.

These results suggest that the photoactivatable prodrug *cis*-[Ru(bpy)_2_(INH)_2_]^2+^ might provide a promising new strategy for combatting antibiotic resistance. Importantly we have shown that this complex is highly selective for killing mycobacteria as opposed to normal human cells. New drugs for combatting mycobacterial infections are in very high demand. Phototherapy using ruthenium complexes such as **1** is likely to be more successful for surface infections where topical administration is facile and the light does not have to penetrate deeply. Hence a target for treatment might be Buruli ulcer, a somewhat neglected and progressively destructive skin disease caused by *Mycobacterium ulcerans* which is endemic in West Africa, China, Australia and spreading.^3*a*^ The huge scale of such infections means that novel approaches to therapy such as that described here are crucial for exploration.

## Experimental section

### Materials

*Cis*-[Ru(bpy)_2_Cl_2_] (bpy = 2,2′-bipyridine) was synthesised as described.^26^ Isoniazid (INH) and NH_4_PF_6_ were purchased from Sigma-Aldrich and used without further purification. The solvents used for UV-visible absorption spectroscopy were reagent grade. The NMR spectroscopy solvent DMSO-d_6_ was purchased from Cambridge Isotope Laboratories Inc and D_2_O was purchased from Sigma-Aldrich. Middlebrook 7H9 Broth Base, glycerol, dextrose, sodium chloride, bovine serum albumin, Tween® 80, phosphate-buffered saline tablets and tryptic soy broth were purchased from Sigma Aldrich. The bacteria, *B. subtilis* 168 and *E. coli* C43 (DE3), were kindly provided by Mrs Anne Smith from the Chemical Biology Laboratory, Department of Chemistry, University of Warwick. The *M. smegmatis* was obtained from the National Collection of Type Cultures (NCTC). The normal lung fibroblast cell line (MRC-5) was purchased from ATCC and cultured in DMEM medium (Gibco) supplemented with FCS (10%; Gibco), penicillin (100 U mL^–1^), and streptomycin (100 mg mL^–1^) in a humidified atmosphere at 37 °C and 5% CO_2_.

### Instrumentation

^1^H NMR spectra were recorded on a Bruker AV-400 spectrometer. Elemental analysis was performed by Exeter Analytical using a CHN/O/S Elemental Analyser (CE440). Positive ion ESI-MS spectra were obtained using an Agilent 6130B single quad coupled to an automated sample delivery system (isocratic Agilent 1100 HPLC without column). LC-HRMS analysis was carried out with a Dionex 3000RS UHPLC coupled to a Bruker MaXis Q-TOF mass spectrometer. UV-visible absorption spectra were recorded on a Varian Cary 300 UV-vis spectrophotometer fitted with an external Varian Cary temperature controller.

### X-ray crystallographic data collection and processing

Crystal data were collected on an Oxford Diffraction Gemini four-circle system with Ruby CCD area detector at ambient temperature (293(2) K). Maximum theta was 70.68 deg. The *hkl* ranges were –24/26, –19/19, –23/17. 14 874 reflections measured, 4773 unique [*R*(int) = 0.0329]. Absorption correction was semi-empirical from equivalent reflections; minimum and maximum transmission factors: 0.56; 1.00.

### Structure analysis and refinement

Systematic absences indicated space group *C*2/*c* or *Cc*. The former was chosen on the basis of intensity statistics and shown to be correct by successful refinement. The structure was solved by direct methods using SHELXS (Sheldrick, 1997) (TREF) with additional light atoms found by Fourier methods. Hydrogen atoms were added at calculated positions and refined using a riding model. Anisotropic displacement parameters were used for all non-H atoms; H-atoms were given isotropic displacement parameters equal to 1.2 times the equivalent isotropic displacement parameter of the atom to which the H-atom is attached.

The asymmetric unit contains half an Ru, a bipy and a pyridylhydrazide, a PF_6_ counter ion and some dioxane solvent. The Ru sits on a two-fold axis. The hydrazide group on the pyridyl was modelled as disordered over two positions. The occupancy factors refined to 50 : 50. The amide hydrogen of these hydrazide groups was placed at a calculated position using AFIX 43. The two hydrogens on the terminal NH_2_ were located in a difference map and refined with DFIX and DANG restraints and given thermal parameters of 1.2 times the equivalent isotropic displacement parameter of the nitrogen to which they are attached. The PF_6_ was modelled as disordered about the central meridian and refined to an occupancy of 59 : 41 major to minor. There were several molecules of dioxane in the crystal. These were very disordered.

Dioxane O20–C25 was modelled at half occupancy. Dioxane O20A–C25A was modelled at 0.25 occupancy and was situated roughly on the same position as dioxane O20–C25. Dioxane O20B–C25B was disordered about a two-fold axis and so was refined at half occupancy under a PART-1 in Shelxl. No hydrogens were placed on the minor component dioxane O20A-C25A but these were included in the formula for completeness. Many DFIX, DANG and SIMU restraints were used to give these disordered components reasonable bond lengths, bond angles and thermal parameters. All the disordered components were refined isotropically. The hydrazine forms an H-bonded dimer with the neighbouring hydrazide of a symmetry-related complex (across inversion centre).

X-ray crystallographic data for **1** have been deposited in the Cambridge Crystallographic Data Centre under the accession number CCDC ; 1474453.

### Light sources

The output of the KiloArc™ and LED light sources was monitored using an International Light Technologies Powermeter (ILT1400-A) equipped with SEL 033 detector and flat response visible filter F/W (400–1064 nm).

The complex was tested against bacteria using the 96-array of blue LEDs (*λ*_irr_ = 465 nm, 20 mW cm^–2^) or the 32-array of multi-coloured LEDs (*λ*_irr_ = 465 nm, 520 nm, 589 nm and 625 nm, 5 mW cm^–2^). The 96- and 32-array multi-colored LEDs were constructed by Mr Rod Wesson, Electrical and Electronics Workshop, Department of Chemistry, University of Warwick. Pictures of the designed LED setups are shown in Fig. S8.† The 96-array LEDs used were Multicomp OVL-5523 5 mm LEDs with a dominant wavelength of 465 nm and a power of 20 mW cm^–2^ once constructed into the array. The 32-array of multi-colored LEDs used were Multicomp OVL-5523 5 mm (dominant wavelength of 465 nm), Multicomp OVL-5524 5 mm (dominant wavelength of 520 nm), Multicomp OVL-5526 5 mm (dominant wavelength of 589 nm) and Multicomp OVL-5528 5 mm (dominant wavelength of 625 nm) with a power of 5 mW cm^–2^ once constructed into the array. The change in optical density (OD) of a bacterial suspension at 600 nm was monitored using Thermo Labsystems IEMS MF microplate reader with a 96-well plate.

### Synthesis of *cis*-[Ru(bpy)_2_(INH)_2_][PF_6_]_2_ (**1**)

*Cis*-[Ru(bpy)_2_(Cl)_2_] (104 mg, 0.2 mmol) and isoniazid (INH) (137 mg, 1 mmol) in 15 mL water were reacted under nitrogen at 353 K for 6 h. The solution was allowed to cool to room temperature and filtered to remove any unreacted starting material. Then NH_4_PF_6_ (163 mg, 1 mmol) was added to give a precipitate. The precipitate was collected by filtration and washed with cold water and diethyl ether. Yield: 66% (130 mg, 0.1 mmol). Elemental analysis calculated for C_32_H_30_F_12_N_10_O_2_P_2_Ru·2H_2_O, %C: 37.92, %H: 3.38, %N: 13.82; found %C: 37.99, %H: 3.07, %N: 13.35. ESI-MS calculated for C_32_H_30_N_10_O_2_Ru [M]^2+^ *m*/*z* 344.1, found *m*/*z* 343.9. ^1^H-NMR (DMSO-*d*_6_, 400 MHz) *δ* H: 4.7 (4H, NH2), 7.5 (2H, t, *J* = 6.4 Hz), 7.6 (4H, d, *J* = 6.7 Hz), 7.9 (2H, t, *J* = 6.5 Hz), 7.9 (2H, d, *J* = 5.1 Hz), 8.0 (2H, t, *J* = 7.5 Hz), 8.2 (2H, t, *J* = 7.3), 8.5 (4H, d, *J* = 6.4 Hz), 8.6 (2H, d, *J* = 8.3 Hz), 8.7 (2H, d, *J* = 7.8 Hz), 9.0 (2H, d, *J* = 5.4 Hz), 10.2 (2H, NH)

Crystals of *cis*-[Ru(bpy)_2_(INH)_2_][PF_6_]_2_·2.5(dioxane) suitable for X-ray crystallography were obtained from slow diffusion of 1,4-dioxane into a saturated acetonitrile solution of **1** at ambient temperature.

### Photoirradiation followed by LC-HRMS

*Cis*-[Ru(bpy)_2_(INH)_2_][PF_6_]_2_ was dissolved in deionised water to give a complex concentration of 40 μM. The solution (400 μL) was placed into a 1 cm path-length quartz cuvette and irradiated using a blue LED light source (*λ*_irr_ = 465 nm, 20 mW cm^–2^) at 298 K for various times. The sample was diluted with deionised water and injected into the LC-HRMS instrument. The mobile phases consisted of A (water with 0.1% trifluoroacetic acid) and B (ACN with 0.1% trifluoroacetic acid). A gradient of 10% B (0 min), 80% B (30 min) was employed with a flow rate of 1 mL min^–1^ and UV detection at 254 nm.

### Photoirradiation followed by ^1^H-NMR spectroscopy

*Cis*-[Ru(bpy)_2_(INH)_2_][PF_6_]_2_ was dissolved in D_2_O to give a complex concentration of ∼6 mM and placed in a 5 mm o.d. NMR tube. The sample was photoirradiated using a blue LED (*λ*_irr_ = 465 nm, 20 mW cm^–2^) at 298 K for various times. The ^1^H-NMR spectrum of each sample was recorded on a Bruker AV-400 spectrometer at 298 K. Data processing was carried out using Bruker Topspin 2.1.

### Photoactivity against bacteria and mammalian cell

The tryptic soy broth (TSB) and phosphate-buffered saline (PBS) were prepared as per manufacturer's instructions. An overnight culture of *E. coli* in TSB was centrifuged and the cell pellet was re-suspended in PBS to give a final OD of 0.015. The *E. coli* suspension was added to wells in a black 96-well plate (100 μL). *Cis*-[Ru(bpy)_2_(INH)_2_][PF_6_]_2_ dissolved in PBS was added to the wells (100 μL) to give final complex concentrations of 200 μM, 100 μM and 10 μM. In the dark and light control wells only PBS (100 μL) was added. Each well was prepared in triplicate. Two black 96-well plates were prepared; one for photoirradiation and one for the dark controls. Both black 96-well plates were incubated initially in the dark for 3 h at 298 K. Subsequently, one of the black 96-well plates was photoirradiated using the 96-array blue LED (*λ*_irr_ = 465 nm, 20 mW cm^–2^) at 298 K for 2 h, while the other black 96-well plate was kept in the dark. The contents of each well (100 μL) were plated onto TSB agar plates and incubated overnight at 310 K. After incubation the number of colony forming units (CFU mL^–1^) were counted. The same procedure was performed using *B. subtilis*. The student's *t*-test was performed with two-tail distribution and unequal variance to determine *p* values (*denotes *p* ≤ 0.05 while **denotes *p* ≤ 0.01); the light samples were compared to the dark samples.

Photoactivity against *M. Smegmatis* using 96-array of blue LEDs and 32-array of multi-colored LEDs. Prior to use, the *M. smegmatis* stock suspension was diluted to OD 0.002 with 7H9 media.^27^ The resulting solution was placed into wells of a black 96-well plate (100 μL). The complex *cis*-[Ru(bpy)_2_(INH)_2_][PF_6_]_2_ was dissolved in 7H9 medium and added to the wells (100 μL) to give various final complex concentrations. In the dark and light control wells only 7H9 medium (100 μL) was added. Each well was prepared in triplicate. Two black 96-well plates were prepared. Both black 96-well plates were incubated initially in the dark for 1 h at 298 K. Subsequently, one of the black 96-well plates was irradiated using the 96-array blue LED (*λ*_irr_ = 465 nm, 20 mW cm^–2^) for various times of irradiation, while the other black 96-well plate was kept in the dark. The contents of both black 96-well plates were transferred to a clear 96-well plate which was placed into a Thermo Labsystems IEMS MF microplate reader. The growth of *M. smegmatis* was monitored by measuring the change in OD at 600 nm at 310 K over 72 h. The percentage survival was calculated by comparing the values obtained to the appropriate control; light exposed samples were compared to the light control and the dark samples were compared to the dark controls. The student's *t*-test was performed with two-tail distribution and unequal variance to determine *p* values (*denotes *p* ≤ 0.05 while **denotes *p* ≤ 0.01). The minimum inhibitory concentration (MIC) is defined at the minimum concentration that produced <5% survival of bacteria. The same procedure as above used the 32-array of multi-colored LEDs (*λ*_irr_ = 465 nm, 520 nm, 589 nm and 625 nm, 5 mW cm^–2^).

Photoactivity against human lung cell MRC-5 was tested using the 96-array of blue LEDs. The normal lung cell MRC-5 solution was placed into two black 96-well plates (100 μL). Complex **1** was dissolved in DMEM medium with 1% DMSO and added to the wells (100 μL) to give various final concentrations. In the dark and light control wells only DMEM medium was added. Each well was prepared in triplicate. Two black 96-well plates were incubated initially in the dark for 1 h in the incubator. Subsequently, one of the black 96-well plates was irradiated using the 96-array blue LED (*λ*_irr_ = 465 nm, 20 mW cm^–2^) for 1 min, while the other black plate was kept in the dark. Then the cells were incubated for another 72 h in the incubator. The cell viability of MRC-5 cells was monitored using a MTT method. The contents of two plates were transferred to clear 96-well plates which were placed into a Promega microplate reader.

### Transient electronic (UV-vis) absorption spectroscopy

A solution of **1** was delivered using a steel flow-through cell (Harrick Scientific), equipped with two CaF_2_ windows and a 950 μm thick Teflon spacer, which defines the optical path length. The sample was recirculated using a peristaltic pump (Masterflex) with PTFE tubing throughout, at a flow speed sufficient to ensure a fresh solution was sampled with each laser shot. Sample was excited using 340 nm, ∼80 fs pump pulses. The pump beam was focused ∼10 mm behind the sample to ensure a beam waist at the sample of ∼250 μm (∼5 times that of the probe) and return pump fluences of 1–2 mJ cm^–2^. The use of a 500 Hz mechanical chopper (Thorlabs) in the pump beam created alternating pumped and non-pumped sample spectra from which a difference spectrum may be calculated. Pump–probe delays (up to 2 ns) were created using a motorized optical delay line in the probe beam path. The pump and probe pulses were generated from a commercially available femtosecond Ti-sapphire regenerative amplified laser system operating at 1 kHz and producing 3 mJ pulses. The output of the laser system (SpectraPhysics, Spitfire XP) was split equally to give three 800 nm beams (1 mJ per pulse each), two of which were used in the present measurements. One of the beams was used to pump an optical parametric amplifier (OPA) (Light-Conversion, TOPAS) to generate 340 nm pump pulses. The second beam was used to generate the broadband white light continuum (340 to 675 nm) probe pulses, by focusing <5% of this 1 mJ per pulse beam into a vertically translated CaF_2_ window. This beam was detected using a fibre coupled UV/vis spectrometer (Avantes, AvaSpec-ULS1650F-USB2). Probe polarisation was held at the magic angle (54.7°) relative to the pump polarisation.

### Computational analysis

All calculations were performed using Gaussian 03. Becke's three-parameter hybrid functional^28^ with the Lee–Yang–Parr's gradient-corrected correlation functional^29^ (B3LYP) was used with LanL2DZ basis set^30^ and effective core potential for the ruthenium atom, and the split valence 6-31G** basis set^31^ for all other atoms. Geometry optimisations of the ground state (*S*_0_) and the lowest-lying triplet state (*T*_1_) were performed in the gas phase and the nature of all stationary points was confirmed by normal mode analysis. For the lowest-lying triplet states, the unrestricted Kohn–Sham method (UKS) was utilised with unrestricted B3LYP functional (UB3LYP). The electronic structure and excited states in solution were calculated using the conductor-like polarisable continuum model method^32^ (CPCM) with water as the solvent. Fifty singlet excited states (with corresponding oscillator strengths) and sixteen triplet excited states (starting from the lowest-lying triplet state geometry) were calculated by time-dependent density functional theory (TD-DFT).^33^ The electronic distribution and localization of the excited states were assigned using GaussSum 2.2.5.^34^

## Conflict of interest

The authors declare no competing financial interests.
