# Supplementary material for: Combatting AMR: photoactivatable ruthenium(ii)-isoniazid complex exhibits rapid selective antimycobacterial activity
Source: Chem Sci. 2016 Aug 30;8(1):395–404. doi: 10.1039/c6sc03028a (PMC5365061; doi:10.1039/c6sc03028a)
Supplement: Supplementary file 1 [file SC-008-C6SC03028A-s001.pdf]

Supporting Information

Combatting AMR: Photoactivatable Ruthenium(II)-isoniazid Complex Exhibits Rapid Selective Antimycobacterial Activity†

Nichola A. Smith,[+]a Pingyu Zhang,[+]a Simon E. Greenough,[a,#] Michael D. Horbury,[a] Guy Clarkson,[a] Daniel McFeely,[b] Abraha Habtemariam,[a] Luca Salassa,[c,d,e] Vasilios G. Stavros,[a] Christopher Dowson,*[b] Peter J. Sadler*[a]

[a]*Department of Chemistry, University of Warwick, Gibbet Hill Road, Coventry CV4 7AL, UK*

[b]*School of Life Sciences, University of Warwick, Gibbet Hill Road, Coventry CV4 7AL, UK*

[c]*CIC biomaGUNE, Paseo de Miramón 182, Donostia-San Sebastián, 20009, Spain*

[d]*Kimika Fakultatea, Euskal Herriko Unibertsitatea and Donostia International Physics Center (DIPC), P.K. 1072, Donostia-San Sebastián, 20080, Spain*

[e]*Ikerbasque, Basque Foundation for Science, Bilbao, 48011, Spain*

[#]Current address: Department of Chemistry, University of Sheffield, Sheffield, S3 7HF, UK

E-mail: P.J.Sadler@warwick.ac.uk; C.G.Dowson@warwick.ac.uk

Table of Contents

Table S1. Crystallographic data for *cis*-$[Ru(bpy)_2(INH)_2][PF_6]_2$……………………..……S3

Table S2. Selected bond lengths (Å) and angles (°) for *cis*-$[Ru(bpy)_2(INH)_2][PF_6]_2$……...S3

Table S3. Experimental extinction coefficients for *cis*-$[Ru(bpy)_2(INH)_2][PF_6]_2$…………..S4

Table S4. TD-DFT calculated vertical singlet transitions for *cis*-$[Ru(bpy)_2(INH)_2][PF_6]_2$..S4

Table S5. Bond distances (Å) for the ground state (S_0) geometry and the lowest-lying triplet state (T_1) geometry of *cis*-$[Ru(bpy)_2(INH)_2]^{2+}$………………………………………..……S4

Figure S1. Solid state structure, X-ray crystal structure connecting two enantiomers in the unit cell and X-ray crystal structure showing hydrogen bonding between the hydrazide group of two neighbouring enantiomers of *cis*-$[Ru(bpy)_2(INH)_2]^{2+}$…………………...…..S5

Figure S2. The dark stability of *cis*-$[Ru(bpy)_2(INH)_2]^{2+}$ for 6 h………...………..……….S6

Figure S3. The extinction coefficient for *cis*-$[Ru(bpy)_2(INH)_2]^{2+}$……….................…....S6

Figure S4. LC-HRMS analysis of *cis*-$[Ru(bpy)_2(INH)_2]^{2+}$ photoirradiated with blue LED for various times…………………………………………………….………………….…..S7

Figure S5. Experimental and calculated HRMS peaks for the photoproduct *cis*-$[Ru(bpy)_2(H_2O)(OH)]^{2+}$..............………….…….............………..…….............………..S8

Figure S6. Change in UV-visible spectrum of *cis*-$[Ru(bpy)_2(INH)_2]^{2+}$ when photoirradiated with blue light for various times and subsequently incubated in the dark for 1 h…………S9

Figure S7. ^{1}H NMR spectrum of *cis*-$[Ru(bpy)_2(INH)_2]^{2+}$ in D_2O before and after 50 min photoirradiation using the blue LED…………………………………..………...……...S10

Figure S8. Images of 96-array of blue LEDs and 32-array multi-colored LEDs ………...S11

Figure S9. The survival of *M. smegmatis* incubated with the complex and then photoirradiated for various times….…………………………………………...…….S11

Figure S10. Photosurvival of *M. smegmatis* (as control) using the 32-array of multi-colored LED..…………………………………………………………………...…….S12

Figure S11. Atom labels for *cis*-$[Ru(bpy)_2(INH)_2]^{2+}$……………..…………………....S12

Figure S12. Experimental absorption spectra and TD-DFT calculated vertical singlet transitions for *cis*-$[Ru(bpy)_2(INH)_2]^{2+}$……………………………………………….S13

Figure S13. Molecular orbitals for the ground state (S_0) geometry of *cis*-$[Ru(bpy)_2(INH)_2]^{2+}$………………………………………………………………..S13

Table S1. Crystallographic data for *cis*-[Ru(bpy)$_2$(INH)$_2$][PF$_6$]$_2$ (**1**.2PF$_6$)

	cis-[Ru(bpy)$_2$(INH)$_2$][PF$_6$]$_2$
Formula	$C_{42}H_{48}F_{12}N_{10}O_7P_2Ru$
Molar mass	1195.91
Crystal system	Monoclinic
Space group	C2/c
Crystal size (mm)	0.20 x 0.08 x 0.04
Crystal character	Orange block
a (Å)	21.9093(7)
b (Å)	15.8193(3)
c (Å)	19.1282(12)
α (°)	90
β (°)	130.884(2)
γ (°)	90
T (K)	293(2)
Z	4
R1 [I>2σ(I)]	0.0798

Table S2. Selected bond lengths (Å) and angles (°) for *cis*-[Ru(bpy)$_2$(INH)$_2$][PF$_6$]$_2$·2.5(dioxane)

Bond length (Å) / angle (°)	*cis*-[Ru(bpy)$_2$(INH)$_2$][PF$_6$]$_2$
Ru1-N1	2.066(5)
Ru1-N12	2.057(4)
Ru1-N13	2.101(4)
N1-Ru-N12	78.62(18)
N12-Ru1-N13	90.26(18)
N1-Ru1-N13	91.3(2)

Table S3. Experimental extinction coefficients for *cis*-[Ru(bpy)$_2$(INH)$_2$][PF$_6$]$_2$ in aqueous solution.

Wavelength (nm)	Extinction Coefficient (M^{-1} cm^{-1})
424	15900
375	17300
288	42300

Table S4. TD-DFT calculated vertical singlet transitions for *cis*-[Ru(bpy)$_2$(INH)$_2$][PF$_6$]$_2$.

Sn	Energy (eV)	*f*	Major contribution	Character
1	2.81 (441)	0.003	HOMO→LUMO (81%)	MLCT(Ru-bpy)
3	2.91 (427)	0.004	H-1→LUMO (79%)	MLCT(Ru-bpy)
5	3.05 (407)	0.092	H-2→L+1 (76%)	MLCT(Ru-bpy)
6	3.09 (401)	0.100	H-2→LUMO (53%)	MLCT(Ru-bpy)
11	3.41 (364)	0.161	H-2→L+2 (78%)	MLLCT(Ru/bpy-INH)
12	3.50 (354)	0.013	HOMO→L+10 (55%)	MC
15	3.62 (343)	0.084	H-2→L+3 (28%)	MC/MLCT(Ru-INH)
45	4.47 (278)	0.364	HOMO→L+9 (37%)	MLCT(Ru-INH)/LC(bpy)

Table S5. Bond distances (Å) for the ground state (S_0) geometry (in comparison to crystal structure) and the lowest-lying triplet state (T_1) geometry of *cis*-[Ru(bpy)$_2$(INH)$_2$]$^{2+}$.

	Bond distance (Å)			
	S_0		T_{MLCT}	T_{MC}
	DFT	X-Ray		
Ru-N_1	2.124	2.066	2.081	2.179
Ru-N_2	2.103	2.057	2.090	2.388
Ru-N_3	2.103	2.057	2.037	2.116
Ru-N_4	2.121	2.066	2.146	2.109
Ru-L_5	2.167	2.101	2.208	3.790
Ru-L_6	2.168	2.101	2.174	2.160

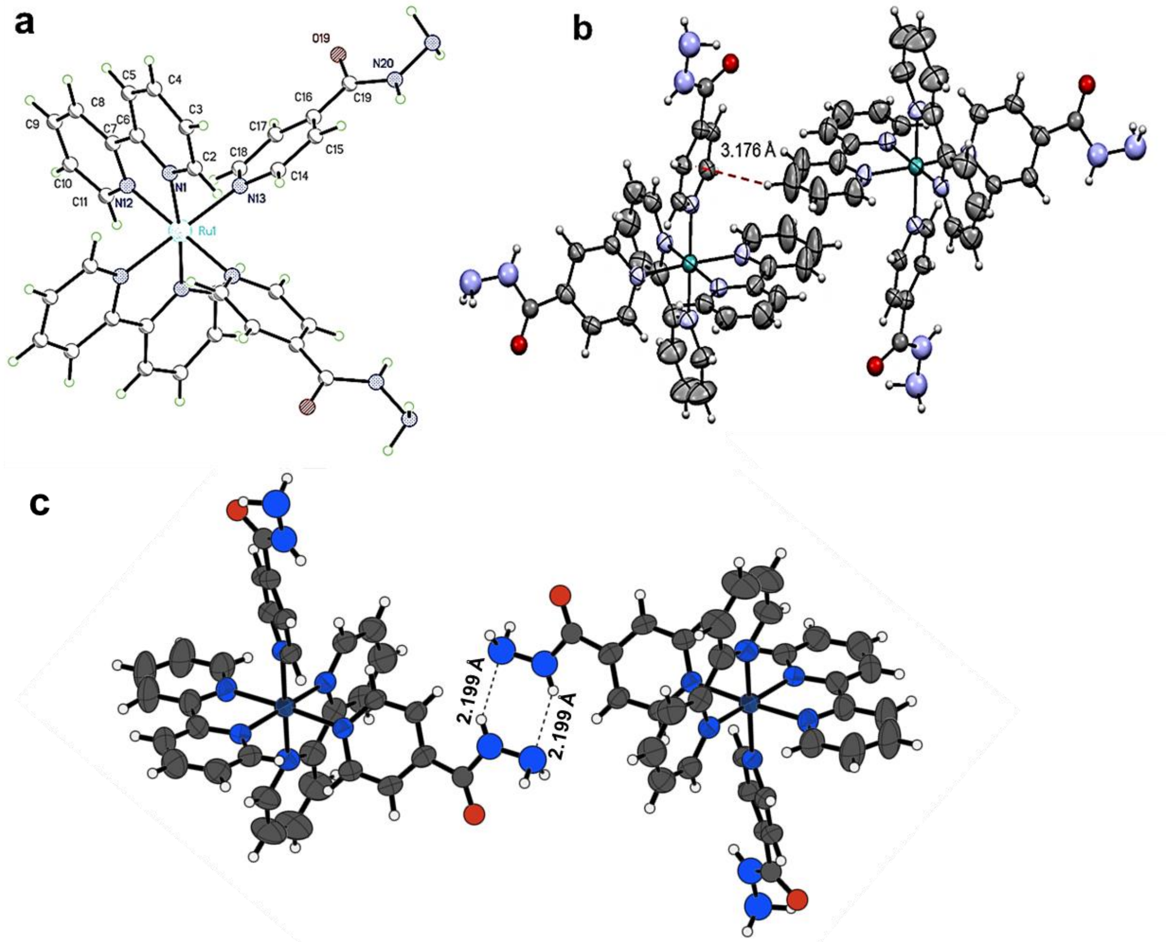

Fig. S1 X-ray crystal structure of *cis*-$[Ru(bpy)_2(INH)_2][PF_6]_2 \cdot 2.5$(dioxane) with counter ions, solvent and minor disordered components removed for clarity. (a) Structure of the cation *cis*-$[Ru(bpy)_2(INH)_2]^{2+}$; (b) CH(bpy)-π(INH) interactions connecting two enantiomers in the unit cell; (c) Hydrogen bonding between the hydrazide groups of two neighbouring enantiomers from different unit cells.

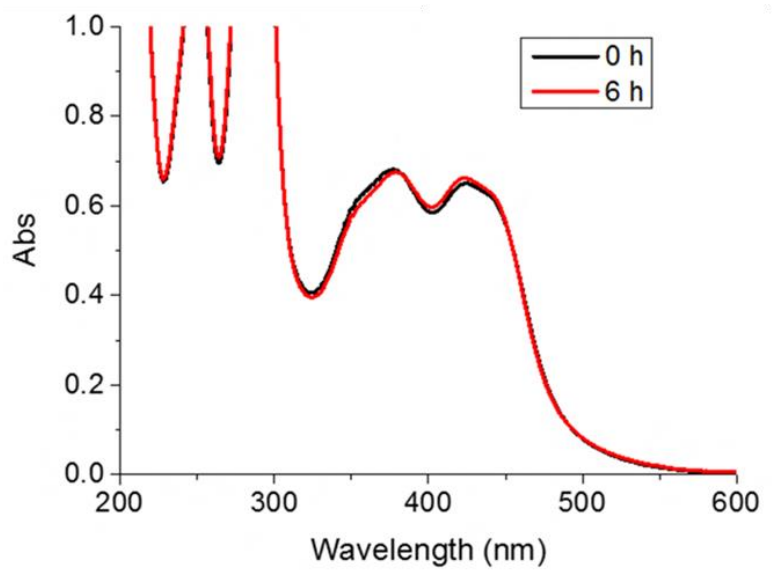

Fig. S2 The dark stability of *cis*-$[Ru(bpy)_2(INH)_2]^{2+}$ for 6 h in water followed by UV-visible spectroscopy.

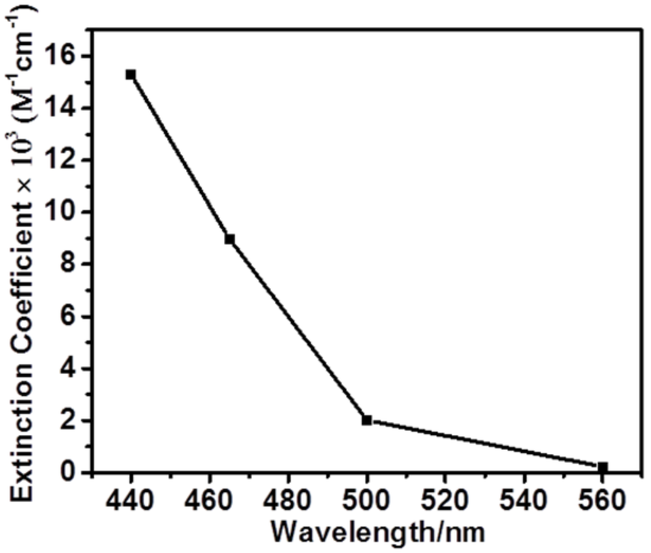

Fig. S3 Extinction coefficients for *cis*-$[Ru(bpy)_2(INH)_2]^{2+}$ at the various wavelengths of photoactivation.

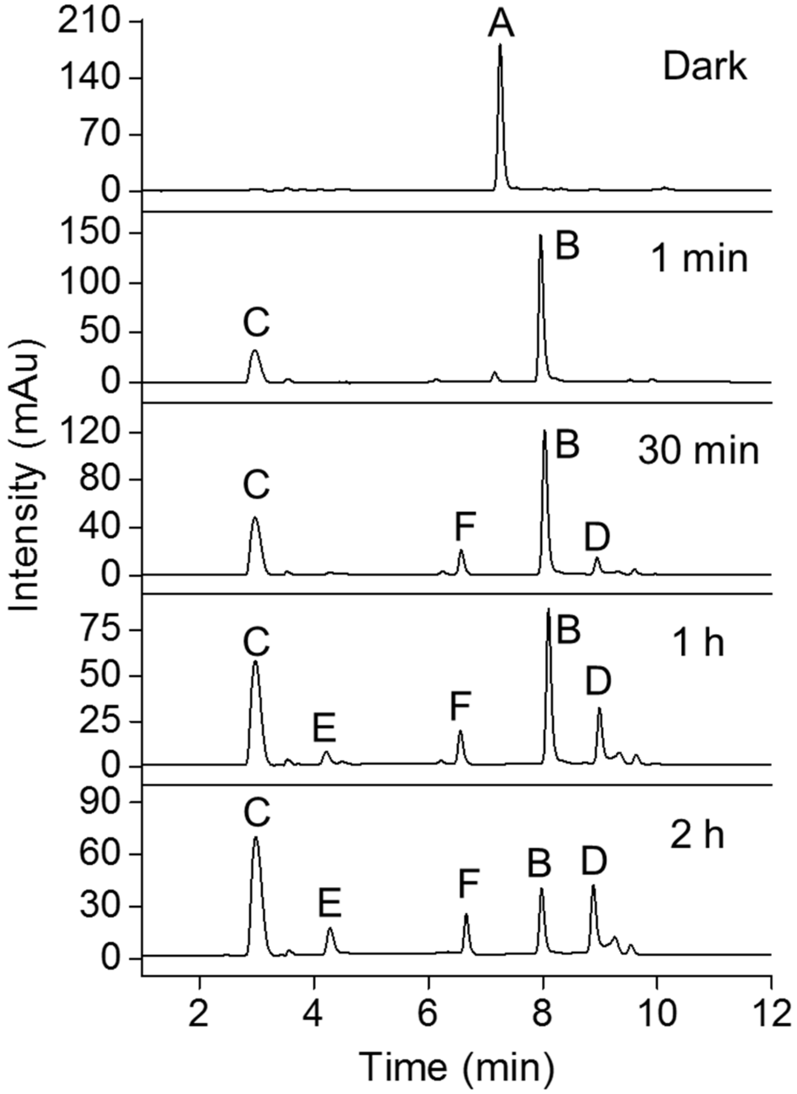

Fig. S4 Chromatograms for *cis*-$[Ru(bpy)_2(INH)_2]^{2+}$ when photoirradiated with blue light (λ_{irr} = 465 nm, 20 mW cm^{-2}) at 298 K for various times; Peak assignments: A: *cis*-$[Ru(bpy)_2(INH)_2]^{2+}$; B: *cis*-$[Ru(bpy)_2(INH)(H_2O)]^{2+}$; C: INH; D: *cis*-$[Ru(bpy)_2(H_2O)_2]^{2+}$; E: *trans*-$[Ru(bpy)_2(H_2O)(OH)]^{2+}$; F: *trans*-$[Ru(bpy)_2(H_2O)_2]^{2+}$. The wavelength of detection was 254 nm.

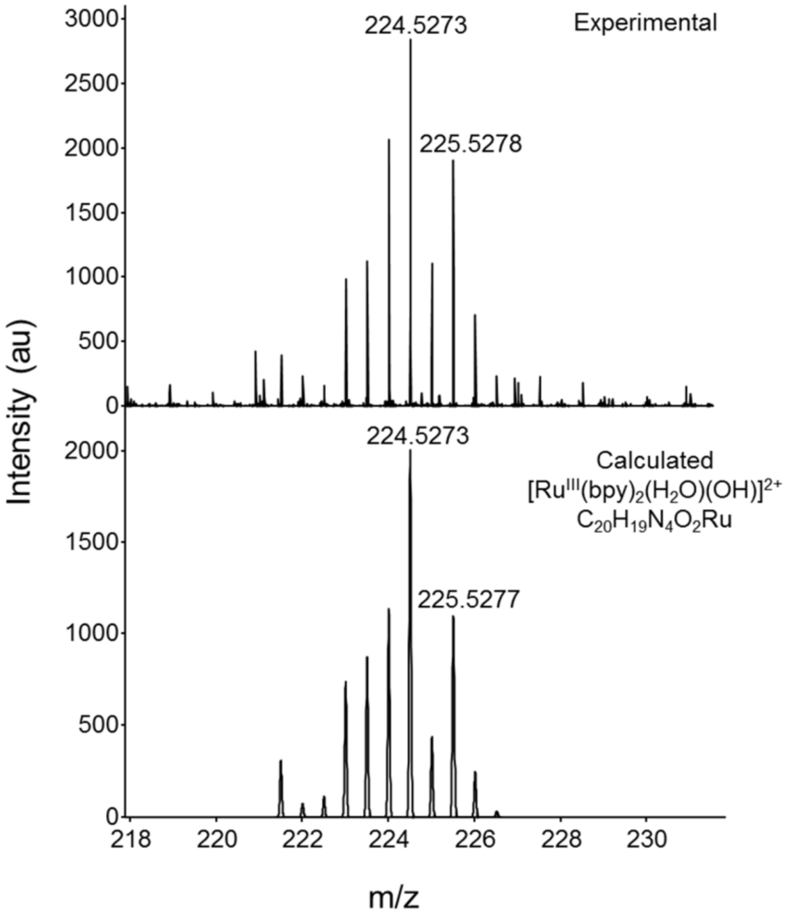

Fig. S5 Experimental and calculated HRMS peaks for the photoproduct *cis*-$[Ru(bpy)_2(H_2O)(OH)]^{2+}$.

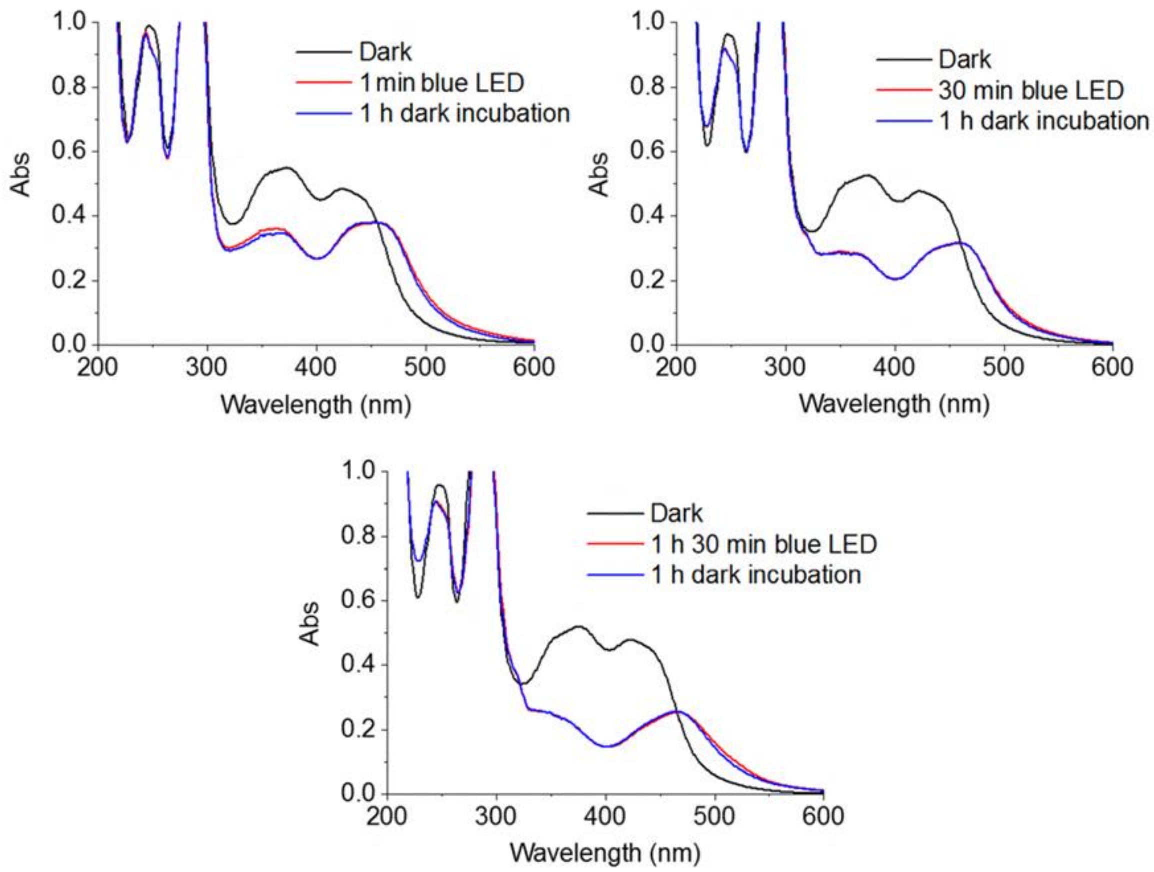

Fig. S6 Change in UV-visible spectrum of *cis*-$[Ru(bpy)_2(INH)_2]^{2+}$ when photoirradiated with blue light (λ_{irr} = 465 nm, 20 mW cm^{-2}) for various times at 298 K and subsequently incubated in the dark for 1 h.

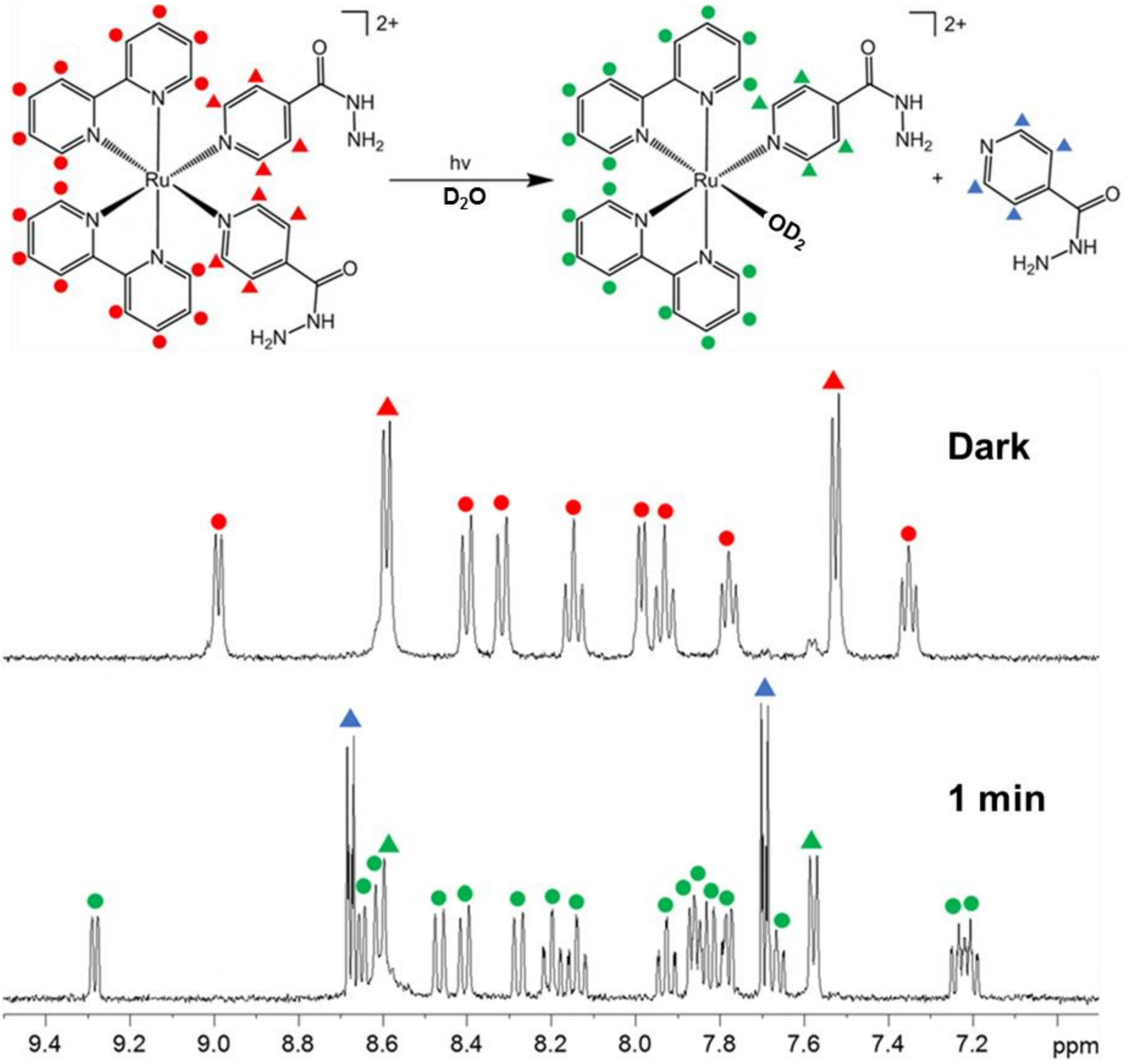

Fig. S7 ^{1}H NMR spectrum (400 MHz) of *cis*-$[Ru(bpy)_2(INH)_2]^{2+}$ in D_2O before (top spectrum) and after 1 min photoirradiation (bottom spectrum) using the blue LED (λ_{irr} = 465 nm, 20 mW cm^{-2}) at 298 K, with schematic of the photoreaction; *cis*-$[Ru(bpy)_2(INH)_2]^{2+}$ = ● (with bound INH ▲), *cis*-$[Ru(bpy)_2(INH)(D_2O)]^{2+}$ = ● (with bound INH ▲), free INH = ▲.

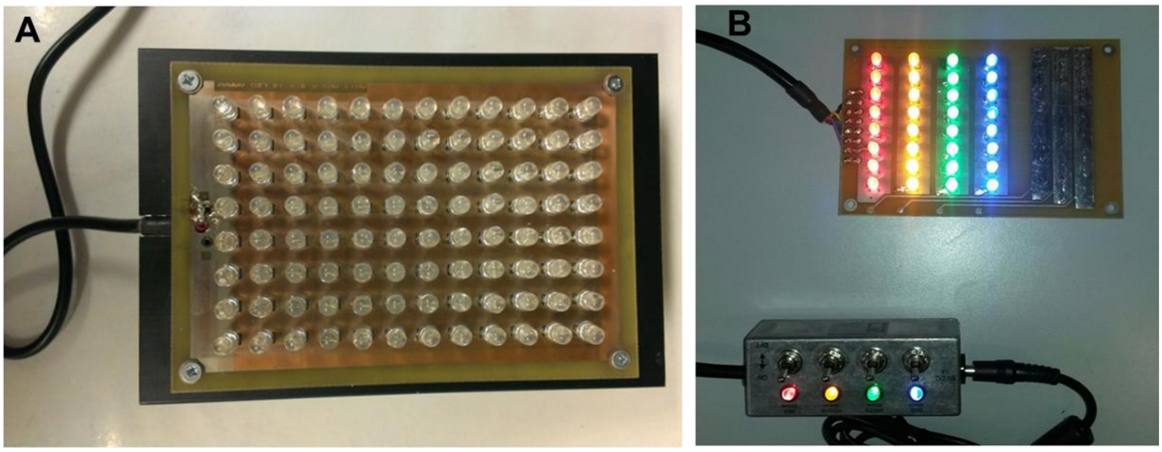

Fig. S8 Images of (A) 96-array of blue LEDs (465 nm, 20 mW cm^{-2}) and (B) 32-array multi-colored LEDs (625 nm, 589 nm, 520 nm and 465 nm, 5 mW cm^{-2}).

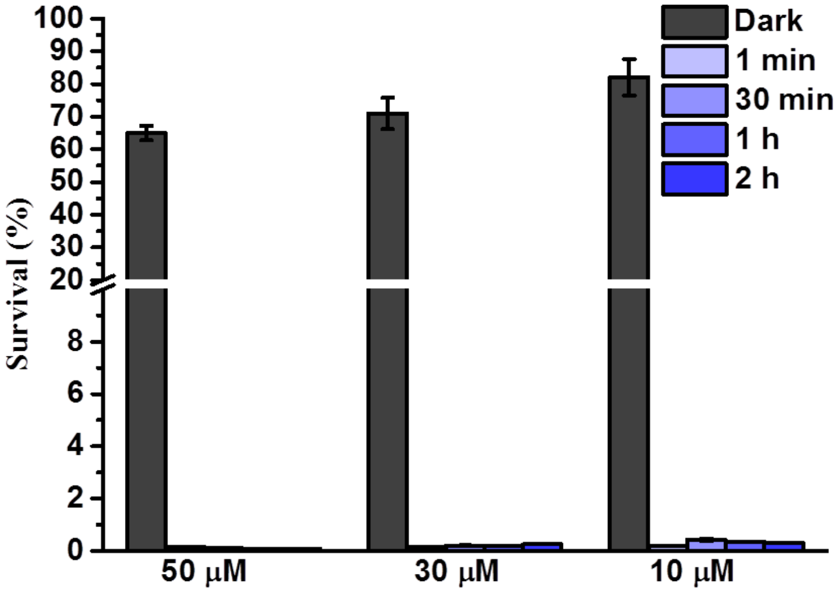

Fig. S9 *M. smegmatis* when incubated with **1** in the dark or photoirradiated using the 96-array blue LED (λ_{irr} = 465 nm, 20 mW cm^{-2}) for various times at 298 K, The survival at each photoirradiation time point was compared to the light control in the absence of the complex, while the survival for the complex in the dark was compared to the dark control in the absence of complex.

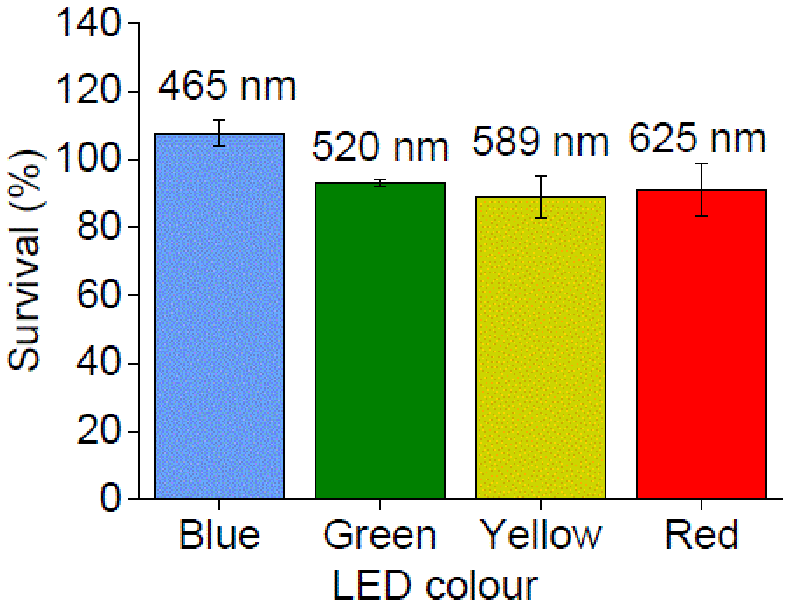

Fig. S10 Photo survival of *M. smegmatis* using the 32-array of multi-colored LEDs (λ_{irr} = 465 nm, 520 nm, 589 nm and 625 nm, 5 mW cm^{-2}) at 298 K for 30 min. *p* values were calculated by comparing the light samples to the dark control and are labelled as follows, $p \leq 0.05$ = *, $p \leq 0.01$ = **.

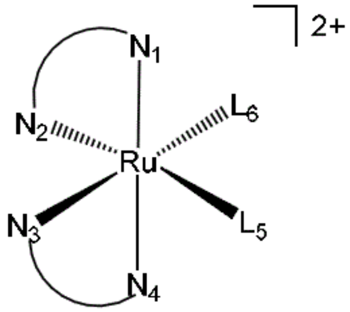

Fig. S11 Atom labels for *cis*-[Ru(bpy)$_2$(INH)$_2$]$^{2+}$, where N_1, N_2, N_3 and N_4 belong to the chelating ligand (*N*-*N'*), and L_5 and L_6 belong to the monodentate ligand (L).

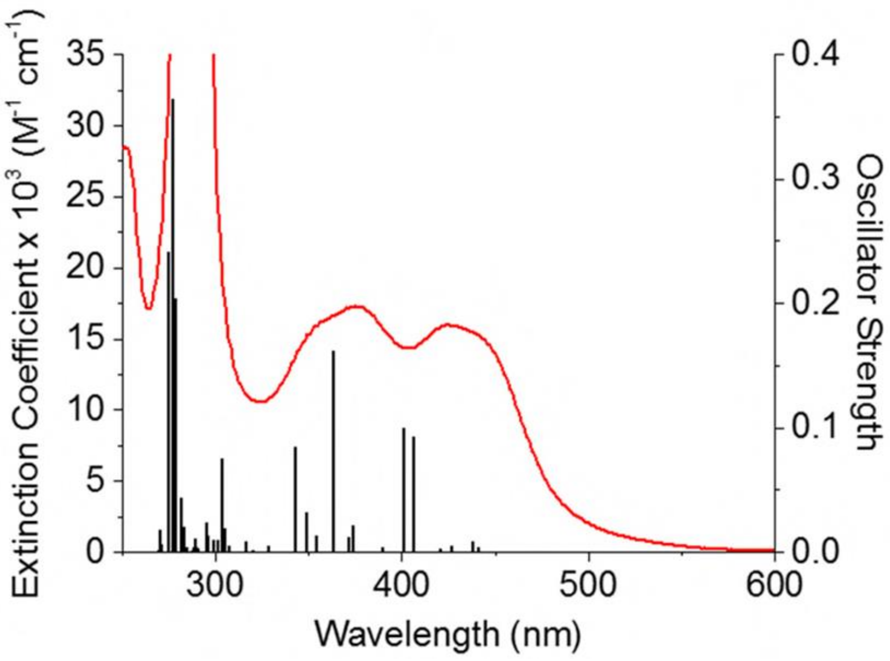

Fig. S12 Experimental absorption spectra (red line, left axis) and TD-DFT calculated vertical singlet transitions (black bars, right axis) for *cis*-$[Ru(bpy)_2(INH)_2]^{2+}$ with water as the solvent.

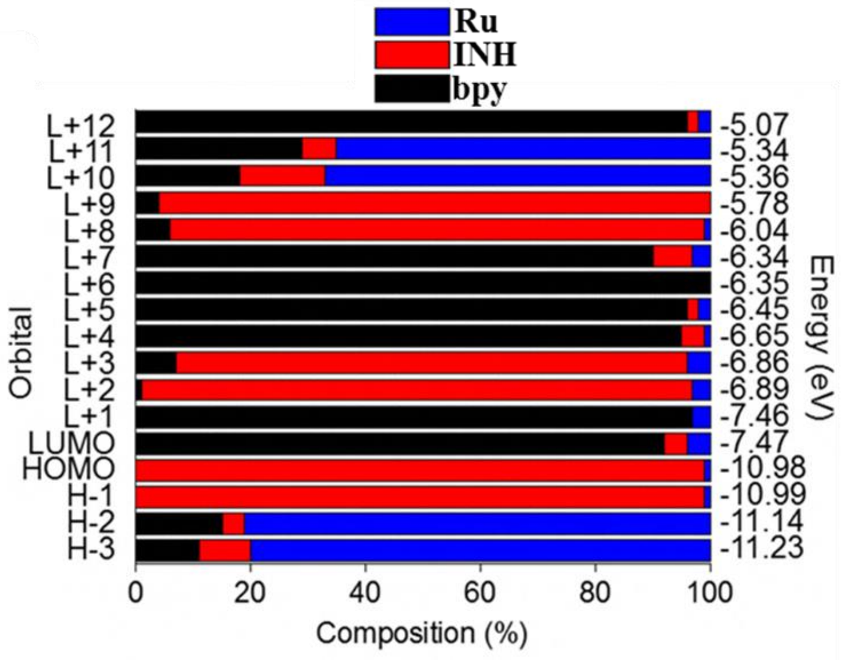

Fig. S13 Composition of selected molecular orbitals for the ground state (S_0) geometry of *cis*- $[Ru(bpy)_2(INH)_2]^{2+}$.
